# Editorial: Incidence, mortality, and risk factors for oral cancers

**DOI:** 10.3389/froh.2025.1636210

**Published:** 2025-06-17

**Authors:** Juliana L. Schussel, Mariana Villarroel-Dorrego, Sven Niklander

**Affiliations:** ^1^ Post Graduation Program in Dentistry, Department of Stomatology, Universidade Federal do Paraná, Curitiba, Brazil; ^2^ Institute of Dental Research, School of Dentistry, Central University of Venezuela Caracas, Caracas, Venezuela; ^3^ Unit of Oral Pathology and Medicine, Dentistry Faculty, Universidad Andres Bello, Viña del Mar, Chile

**Keywords:** cancer incidence and mortality, oral cancer, oral screening, Global Burden Disease, prevention

**Editorial on the Research Topic**
Incidence, mortality, and risk factors for oral cancers

Oral cancer remains a significant global health concern, with a great impact on morbidity, mortality, and quality of life. The Research Topic “*Incidence, Mortality, and Risk Factors for Oral Cancers*” published in Frontiers in Oral Health brings together a collection of studies that provide valuable updates on the epidemiological landscape of oral cancer, uncovering emerging trends, persistent challenges, and potential strategies for prevention and control (1).

According to the most recent Global Burden of Disease (GBD) study (2019), oral cancer presents a multifaceted epidemiological scenario. While age-standardized incidence rates (ASIR) have continued to increase globally, age-standardized mortality rates (ASMR) and disability-adjusted life years (DALYs) have shown a gradual decline, largely due to improvements in early diagnosis and treatment modalities (1). However, these advances are limited to high-income countries, revealing important disparities between regions and socioeconomic groups.

One of the articles in this Research Topic explored the epidemiology of oral squamous cell carcinoma (OSCC) in Europe (Ghanem et al.), identifying notable differences across regions, sexes, and age groups. The article emphasizes the need to address region-specific risk factors, improve early diagnosis through public awareness and healthcare efficiency, and advance personalized treatment strategies, including targeted therapies and radioimmunotherapy. This study also highlights the critical role of HPV in OSCC pathogenesis and calls for further investigation into the biological, behavioral, and social determinants of the disease to inform more effective prevention approaches.

By contrast, a study from southwestern Iran found that demographic variables— such as age, gender, marital status, and socioeconomic indicators—did not significantly affect the 5-year survival rate of OSCC patients (Karimi et al.). The authors advocate for future research to focus instead on pathological features such as recurrence, metastasis, treatment type, and tumor characteristics to better understand and improve patient outcomes.

Globally, men continue to represent the majority of oral cancer cases. However, a growing incidence among women suggests evolving exposure to traditional risk factors such as tobacco and alcohol. This trend underscores the need for gender-sensitive public health strategies (1). Low- and middle-income countries (LMICs) face a disproportionate burden due to late-stage diagnoses, limited treatment capacity, and preventable deaths (2). Low health literacy, restricted access to healthcare services, and a shortage of trained professionals for early detection are some of the obstacles encountered. The absence of specialized diagnostic centers further delays timely intervention. Nonetheless, existing evidence confirms that trained clinicians can accurately assess the clinical risk of oral potentially malignant disorders (OPMDs), making such training a cornerstone of successful screening efforts.

From 1990 to 2019, oral cancer incidence, mortality, and DALYs increased with age. Among individuals under 20, high-income countries saw a paradoxical rise in incidence, alongside a concurrent decline in deaths and DALYs. Conversely, LMICs reported rising mortality in this age group, highlighting serious deficiencies in healthcare infrastructure and our understanding of disease behavior in younger populations (3). Among the elderly—particularly those patients aged 70 to 79—mortality from certain head and neck cancers has increased substantially. While men had a higher number of deaths before the age of 35, DALY trends after the age of 70 revealed a marked decline in mortality in men and a stabilization in women, suggesting sex-based disparities in survival and healthcare access (1).

Tobacco and alcohol remain the principal risk factors for oral cancer. High-income countries have successfully implemented tobacco control measures, whereas LMICs continue to struggle with the influence of the tobacco industry. The rise in e-cigarette use, especially among adolescents, adds a layer of complexity to the issue. These products are aggressively marketed using appealing flavors and misleading health claims, which can create a false sense of safety. Similarly, alcohol companies exploit weak regulations in LMICs by saturating markets with inexpensive, high-alcohol-content beverages that stimulate consumption behaviors.

A study in this Research Topic focusing on rural women who use a hookah, highlighted low to average scores in terms of knowledge, attitudes, and prevention behaviors related to oral cancer (Mohammadkhah et al.). Nicotine dependence was high and was found to be significantly associated with reduced preventive behaviors. These findings, which are grounded in the Theory of Planned Behavior, call for tailored health education programs, particularly in underserved communities. Using mass media and social platforms may enhance outreach, while addressing nicotine addiction remains a critical barrier to effective prevention.

HPV vaccination—a proven strategy for reducing oropharyngeal cancer—faces implementation barriers, especially in resource-limited settings. Access to HPV vaccines is a matter of deep concern and reflects a marked inequity in global health. In resource-limited countries, health systems often must prioritize combating infectious diseases and infant mortality, which can relegate HPV vaccination to a lower priority.

At the same time, advancements in genomics and proteomics have the potential to improve risk stratification and guide individualized surveillance approaches. However, the cost of proteomic analysis, reagents, and highly trained personnel is an insurmountable barrier for the majority of research centers and hospitals. This severely limits the possibility of conducting genomic and proteomic studies locally or incorporating these techniques into clinical practice.

On the other hand, oral cancer screening campaigns in LMICs face significant challenges that limit their impact on disease incidence. While early detection is crucial for improving treatment outcomes and survival rates, several socioeconomic and infrastructural factors hinder the effectiveness of these campaigns in resource-limited settings. When healthcare infrastructure is fragile, with few trained healthcare professionals and few healthcare centers equipped to conduct screening and follow-up, it is difficult for campaigns to reach the target population and for detected cases to receive timely treatment.

It is also important to understand that cancer survivors face life-altering consequences, including disfigurement from radical surgeries and a lack of access to rehabilitation services. Addressing these human costs requires resolute action to prioritize equitable access to care and to integrate oral cancer control into broader public health frameworks.

## Conclusion

While progress has been made, disparities persist. Only through innovation, global collaboration, and a steadfast commitment to health equity can we achieve meaningful reductions in the global burden of oral cancer.

## References

[1] Sun R Dou W Liu W Li J Han X Li S Global, regional, and national burden of oral cancer and its attributable risk factors from 1990 to 2019. Cancer Med. (2023) 12(12):13811–20. 10.1002/cam4.6025 37132165 PMC10315711

[2] Martínez-Ramírez J Saldivia-Siracusa C González-Pérez L-V Zelaya FJMC Gerber-Mora R Cabrera OFG Barriers to early diagnosis and management of oral cancer in Latin America and the Caribbean. Oral Dis. (2024) 30(7):4174–84. 10.1111/odi.14903 38380784

[3] GBD 2019 Lip, Oral, and Pharyngeal Cancer Collaborators, Cunha ARD Compton K Xu R Mishra R Drangsholt MT The global, regional, and national burden of adult lip, oral, and pharyngeal cancer in 204 countries and territories: a systematic analysis for the global burden of disease study 2019. JAMA Oncol. (2023) 9(10):1401–16. 10.1001/jamaoncol.2023.2960 37676656 PMC10485745

